# Phylogeographic Analysis of HIV-1 Subtype C Dissemination in Southern Brazil

**DOI:** 10.1371/journal.pone.0035649

**Published:** 2012-04-18

**Authors:** Gonzalo Bello, Paolo M. de A. Zanotto, Atila Iamarino, Tiago Gräf, Aguinaldo R. Pinto, José C. Couto-Fernandez, Mariza G. Morgado

**Affiliations:** 1 Laboratório de AIDS & Imunologia Molecular, Instituto Oswaldo Cruz, FIOCRUZ, Rio de Janeiro, Brazil; 2 Departamento de Virologia, IMPPG, Universidade Federal do Rio de Janeiro, Rio de Janeiro, Brazil; 3 Laboratório de Evolução Molecular e Bioinformática (LEMB), Departamento de Microbiologia, Instituto de Ciências Biomédicas, Universidade de São Paulo, São Paulo, Brazil; 4 Departamento de Microbiologia, Imunologia e Parasitologia, Universidade Federal de Santa Catarina, Florianópolis, Brazil; National HIV and Retrovirology Laboratories, Canada

## Abstract

The HIV-1 subtype C has spread efficiently in the southern states of Brazil (Rio Grande do Sul, Santa Catarina and Paraná). Phylogeographic studies indicate that the subtype C epidemic in southern Brazil was initiated by the introduction of a single founder virus population at some time point between 1960 and 1980, but little is known about the spatial dynamics of viral spread. A total of 135 Brazilian HIV-1 subtype C *pol* sequences collected from 1992 to 2009 at the three southern state capitals (Porto Alegre, Florianópolis and Curitiba) were analyzed. Maximum-likelihood and Bayesian methods were used to explore the degree of phylogenetic mixing of subtype C sequences from different cities and to reconstruct the geographical pattern of viral spread in this country region. Phylogeographic analyses supported the monophyletic origin of the HIV-1 subtype C clade circulating in southern Brazil and placed the root of that clade in Curitiba (Paraná state). This analysis further suggested that Florianópolis (Santa Catarina state) is an important staging post in the subtype C dissemination displaying high viral migration rates from and to the other cities, while viral flux between Curitiba and Porto Alegre (Rio Grande do Sul state) is very low. We found a positive correlation (*r* ^2^ = 0.64) between routine travel and viral migration rates among localities. Despite the intense viral movement, phylogenetic intermixing of subtype C sequences from different Brazilian cities is lower than expected by chance. Notably, a high proportion (67%) of subtype C sequences from Porto Alegre branched within a single local monophyletic sub-cluster. These results suggest that the HIV-1 subtype C epidemic in southern Brazil has been shaped by both frequent viral migration among states and *in situ* dissemination of local clades.

## Introduction

Through June 2011, about 610,000 cases of AIDS have been cumulatively reported in Brazil since the first identification of AIDS in the early 1980s [1]. The prevailing HIV-1 genetic variants in most country regions are subtypes B, F1 and BF1 recombinants [2], [3], [4], [5], [6]. States from the south region (Rio Grande do Sul, Santa Catarina and Paraná), which concentrate 20% of the Brazilian AIDS cases [1], display a distinct molecular epidemiology profile characterized by the high prevalence of subtypes C, B and BC recombinants. It has been estimated that subtype C represents around 20–30% of HIV-1 infections in Paraná [3], [7], [8], [9], [10], 30–45% of HIV-1 infections in Rio Grande do Sul [3], [11], [12], [13], [14], [15], and 50–80% of HIV-1 infections in Santa Catarina [14], [16], [17], [18].

Phylogeographic studies indicate that the HIV-1 subtype C epidemic in southern Brazil was initiated by the introduction of a single founder virus population probably originated from East Africa [19], [20]. Another study suggested that the United Kingdom (UK) may have played a crucial role in such dissemination, acting as a staging post between Africa and South America [21]. A more recent phylogeographic analysis, however, found no evidence of viral flow from the UK to Brazil, only from East Africa and Brazil to the UK [22]. Thus, the exact route of migration of HIV-1 subtype C from East Africa to Brazil remains unclear. The precise time-scale of such an event is also uncertain. Two independent studies estimated the onset date of Brazilian subtype C epidemic at around the early 1980s [19], [21], while another study suggests that this Brazilian epidemic could be much older, dating back to between 1960 and 1970 [22].

Although several studies have explored the origin and time-scale of the HIV-1 subtype C epidemic in Brazil, there is little information about the spatial dynamic of dispersion of this clade in the country. The only phylogeographic study points toward Paraná or Rio Grande do Sul states as the possible entrance points of HIV-1 subtype C and further indicate an asymmetrical net viral flow following a north to south axis [22]. According to that study Paraná is the hub of the epidemic from where the subtype C is spreading to Santa Catarina and Rio Grande do Sul. That study, however, was based on the analysis of *pol* datasets that included a very small number of sequences (*n* = 14) per state, which may bias the results. In the present study, we reevaluated the spatial pattern of HIV-1 subtype C dissemination in southern Brazilian states, based on the analysis of a larger subtype C *pol* data set containing sequences collected at the capital cities of Rio Grande do Sul (*n* = 55), Santa Catarina (*n* = 41), and Paraná (*n* = 39).

## Materials and Methods

### HIV-1 subtype C Brazilian sequences

**Figure 1:**
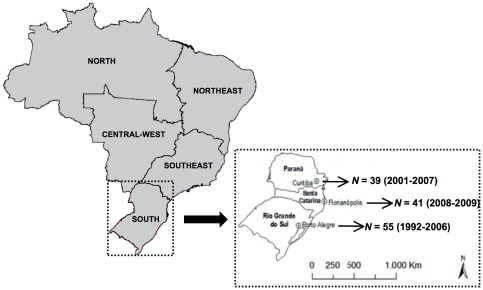
Map of Brazil showing the five country regions. An expanded map of the South region with the states (Paraná, Santa Catarina and Rio Grande do Sul) and state capitals (Curitiba, Florianópolis and Porto Alegre) is shown to the right. The number and sampling dates of sequences from the three southern state capitals included in the present study are indicated.

New protease and partial reverse transcriptase (PR/RT) subtype C Brazilian sequences were obtained from 41 HIV-infected antiretroviral therapy-naïve patients from Florianópolis (capital of the Santa Catarina state) and 18 HIV-infected treated-patients from Curitiba (capital of the Paraná state). Patients from Florianópolis were followed up at the “Hospital Homero de Miranda Gomes", had their samples taken from 2008 to 2009 and were described in more detail by Gräf *et al* [18]. Patients from Curitiba were followed at outpatient clinics from the Public Health System and underwent HIV genotyping tests at the Laboratory of AIDS and Molecular Immunology (FIOCRUZ) between 2006 and 2007, as previously described [23]. These sequences were combined with PR/RT subtype C sequences isolated from antiretroviral therapy-naive patients from Porto Alegre (capital of the Rio Grande do Sul state, *n* = 55) and Curitiba (*n* = 21) described elsewhere [7], [19] (Fig. 1). The study was approved by the Universidade Federal de Santa Catarina and FIOCRUZ Ethics Committees.

### HIV-1 subtype C reference sequences

A reference set of 31 subtype C *pol* gene sequences of African origin was obtained from the Los Alamos HIV Sequence Database (www.hiv.lanl.gov). The reference set includes subtype C sequences representative of the East (Burundi = 5, Djibouti = 1, Ethiopia = 3, Kenya = 2, Somalia = 1, Tanzania = 2, and Uganda = 1), Southern (Botswana = 1, Malawi = 1, South Africa = 4, Zambia = 4, and Zimbabwe = 2), Central (Democratic Republic of Congo = 2, Gabon = 1), and West (Senegal = 1) African regions, sampled over a time interval of 18 years (1986–2004). Among those subtype C references we included some sequences from Burundi, Kenya, and Ethiopia that were described as the ones most closely related to the Brazilian subtype C clade [19], [20].

### Alignment and phylogenetic analysis

Sequences were aligned using CLUSTAL X program [24]. In order to avoid any bias on the phylogenetic analyses, all sites with major antiretroviral drug resistance mutations in PR (50, 82 and 90) and RT (41, 67, 70, 98, 103, 106, 179, 184, 190, 215 and 219) in at least two sequences were excluded, leaving a final alignment of 951 nucleotides (nt) long (covering nt 2253–3272 relative to HXB2 clone). Alignment is available from the authors upon request. The phylogenetic tree was inferred by the maximum likelihood (ML) method under the GTR+I+G nucleotide substitution model, selected using the jModeltest program [25]. ML tree was reconstructed with program PhyML [26] using an online web server [27]. Heuristic tree search was performed using the SPR branch-swapping algorithm and the reliability of the obtained topology was estimated with the approximate likelihood-ratio test (*aLRT*) [28] based on the Shimodaira-Hasegawa-like procedure.

### Analysis of metapopulation structure

The hypothesis of restricted phylogenetic mixing of HIV-1 subtype C sequences obtained from different cities in Southern Brazil was tested using program BaTS [29]. BaTS estimates phylogeny-trait associations using the Association Index (AI) [30] and the Parsimony Score (PS) [31] statistics whilst accounting for phylogenetic uncertainty by the use of the posterior distribution of trees arising from earlier Bayesian Markov Chain Monte Carlo (MCMC) analyses. Bayesian phylogenetic trees were inferred under the GTR+I+G nucleotide substitution model using program MrBayes [32], running for each alignment one MCMC for 30–50×10^6^ generations with a burn-in of 3–5×10^6^ generations. Adequate chain mixing was checked by calculating the effective sample size (ESS) using program TRACER v1.4 [33] after excluding an initial 10% for each run. All parameter estimates showed ESS values >400.

### Analysis of spatial dispersion pattern

Ancestral reconstruction of the locations at the interior nodes of a time-scale phylogenetic tree was obtained using the Bayesian statistical framework implemented in BEAST 1.6.2 [34], [35]. A matrix of geographic locations was constructed based on the city of sampling for each sequence (*n* = 3) and a discrete phylogeographic model was used in which all three possible reversible exchange rates between locations were equally likely (flat prior) [36]. We used a fixed substitution rate (1.5×10^−3^ substitutions/site/year) equal to the mean rate previously estimated for the Brazilian subtype C clade at *pol* gene [19]. Analyses were performed using the GTR+I+Γ_4_ nucleotide substitution model, an uncorrelated Lognormal relaxed molecular clock model [37], and a Bayesian Skyride coalescent model [38]. The MCMC analysis was run until evidence of proper mixing (ESS>200) was obtained. We also used the *BayesTraits* program [39] to estimate posterior probabilities (PP) of root positions and the rates of migration among localities using the set of plausible trees sample at stationary during the MCMC run with BEAST. The programs TreeAnnotator v1.6.2 [35] and FigTree v1.3.1 [40] were used to summarize the posterior tree distribution and to visualize the annotated maximum clade credibility (MCC) tree, respectively.

## Results

### Phylogeographical structure of HIV-1 subtype C epidemic in southern Brazil

**Figure 2:**
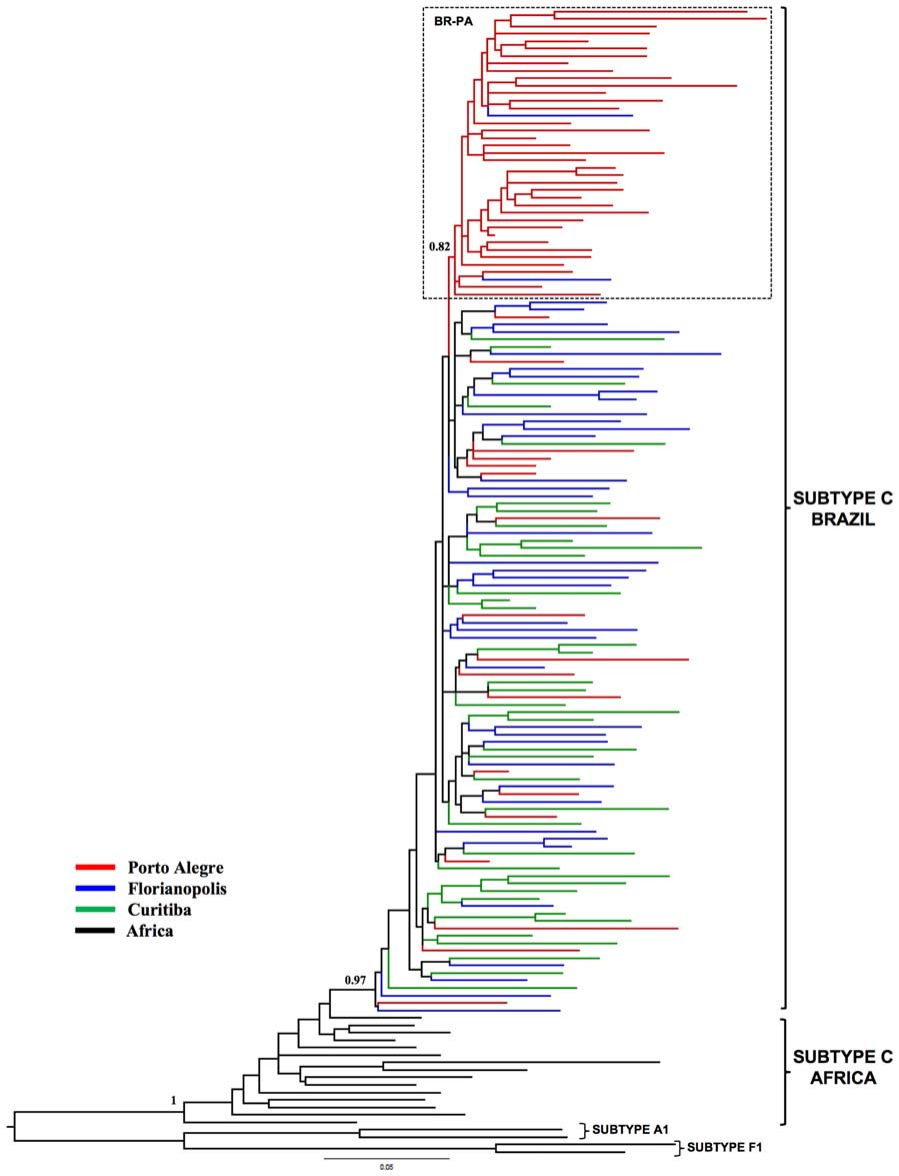
ML phylogenetic tree for HIV-1 subtype C *pol* (PR/RT) sequences circulating in Brazil. The color of a branch represents the geographic region from where the subtype C strain was sampled, according to the legend given in the figure. Brackets indicate the monophyletic clade formed by Brazilian subtype C sequences and the position of subtype C reference sequences of African origin. The box highlights the position of the sub-cluster circulating in Porto Alegre (BR-PA). The *aLRT* support values are indicated only at key nodes. Horizontal branch lengths are drawn to scale with the bar at the bottom indicating nucleotide substitutions per site. The tree was rooted using HIV-1 subtype A1 and F1 reference sequences.

**Figure 3:**
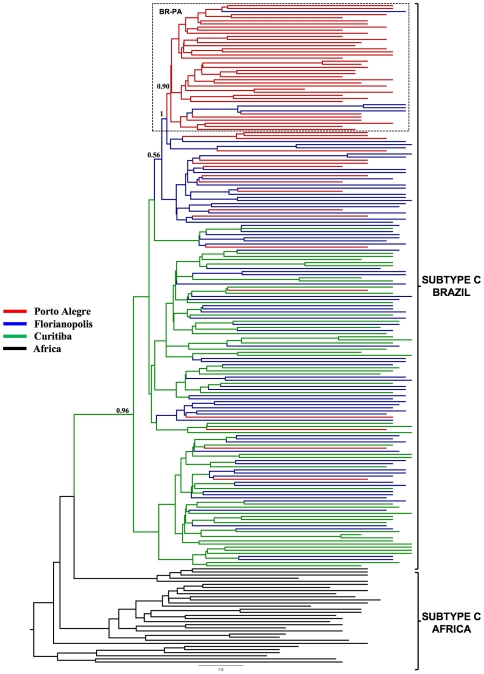
Bayesian MCC tree for HIV-1 subtype C *pol* (PR/RT) sequences circulating in Brazil. Branches are colored according to the most probable location state of their descendent nodes. The legend for the colors is shown on the left. Brackets indicate the monophyletic clade formed by subtype C sequences sampled from Brazil, and the position of subtype C reference sequences of African origin used to root the tree. The box highlights the position of the sub-cluster BR-PA. The state posterior probability is indicated only at key nodes. Horizontal branch lengths are drawn to scale with the bar at the bottom indicating years.

A total of 135 Brazilian HIV-1 subtype C *pol* (PR/RT) sequences from Porto Alegre (*n* = 55), Florianópolis (*n* = 41), and Curitiba (*n* = 39) were analyzed together with some subtype C reference sequences of African origin, including those sequences from East Africa that were described as the ones most closely related to the Brazilian subtype C lineage. ML and Bayesian phylogenetic analyses revealed that all Brazilian subtype C sequences formed a highly supported (*aLRT* = 1, *PP* = 1) monophyletic group (Figs. 2 and 3), thus confirming that the subtype C epidemic in Brazilian southern states was the result of a single founder event, followed by subsequent local expansion.

The ML phylogenetic tree displayed in Fig. 2 showed some level of phylogenetic intermixing of Brazilian subtype C sequences from different geographic locations. Many sequences show no grouping according to their geographic origin, while others fell within small state-specific monophyletic clades of only 2–3 sequences, pointing to relatively frequent virus movement between major southern Brazilian cities. Some degree of phylogeographic subdivision, however, is also apparent in the Brazilian subtype C phylogeny. Notably, a high proportion (67%) of subtype C sequences from Porto Alegre branch within a single monophyletic cluster (*aLRT* = 0.80), here called BR-PA, that was almost exclusively composed by sequences isolated in that state (Fig. 2).

To assess the overall degree of spatial admixture and geographical structure among HIV-1 subtype C lineages in this region, we used the program BaTS to calculate the PS and AI statistics for the posterior distribution of trees arising from Bayesian MCMC analyses of sequences from: Porto Alegre/Florianópolis (PA/FL), Porto Alegre/Curitiba (PA/CU), and Florianópolis/Curitiba (FL/CU). The null hypothesis of panmixis (i.e., complete intermixing of sequences from different Brazilian cities) was rejected by both the AI and PS statistics for all comparisons (Table 1), demonstrating that the geographic subdivision of Brazilian HIV-1 subtype C strains is higher than expected by chance. Thus, the observed HIV-1 subtype C diversity in the south region of Brazil seems to be shaped by both viral migration and viral *in situ* evolution.

**Table 1:** Bayesian MCMC test of phylogenetic isolation of Brazilian HIV-1 subtype C sequences by geographic region.

Geographic regions	Statistic	Observed value (95% CI)	Expected value^a^ (95% CI)	*P*-value
PA-FL	AI	2.98 (2.23–3.73)	5.04 (4.12–5.98)	<0.001
	PS	18.85 (16.00–21.00)	30.61 (27.48–33.35)	<0.001
PA-CU	AI	1.79 (1.14–2.48)	4.83 (3.91–5.64)	<0.001
	PS	11.98 (9.0–15.0)	29.89 (26.88–32.41)	<0.001
FL-CU	AI	2.96 (2.18–3.77)	4.58 (3.60–5.49)	<0.001
	PS	21.39 (19.00–24.00)	26.74 (24.24–29.40)	<0.001

a Expected AI or PS value under the null hypothesis of no phylogenetic clustering of isolates by sampling location. PA: Porto Alegre. FL: Florianópolis. CU: Curitiba.

### Spatial dynamics of HIV-1 subtype C spread in southern Brazil

Having established the existence of statistically significant geographic subdivision as well as evidence of frequent migrations of Brazilian subtype C strains, we applied a Bayesian phylogeographic approach in order to investigate how this structure was established and to estimate the net viral flow among southern states. The overall topology of the Bayesian MCC tree obtained with BEAST corroborates the monophyletic nature of the Brazilian subtype C clade and the existence of the BR-PA sub-clade circulating in Porto Alegre (Fig. 3). Ancestral reconstruction of the locations at the interior nodes of Bayesian tree using both BEAST and BayesTraits further revealed that the most probable place at the root of Brazilian subtype C lineage is Curitiba (*PP*≥0.96) and that the ancestor of the BR-PA clade probably originated in Florianópolis (*PP* = 1).

**Figure 4:**
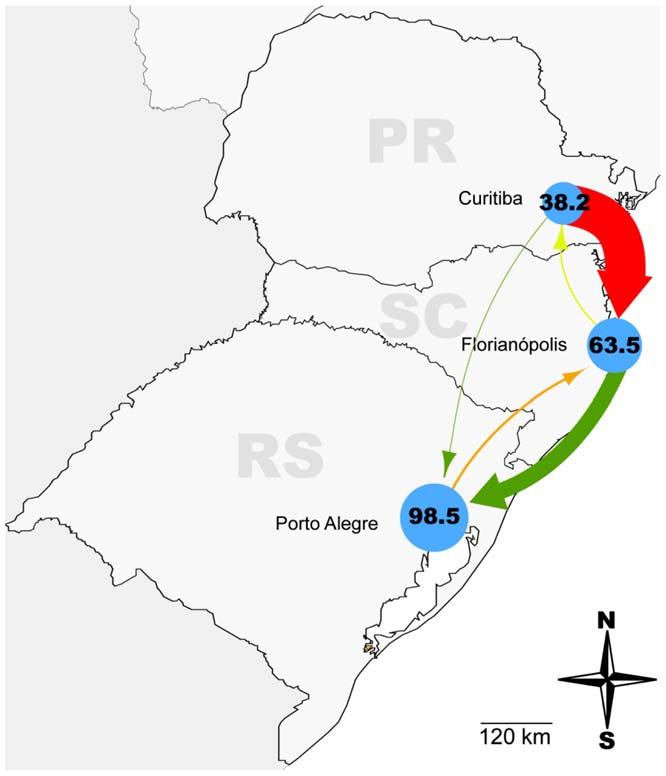
HIV-1 subtype C migration rates and routine traffic of people amongst the three states of the South Brazilian region. Capitals are represented by blue circles proportional to the incidence of AIDS cases per 100,000 inhabitants in each city in 2009 [1]. The arrows are colored according to the routine traffic between states [41], from green (less) to red (more) passing through yellow, and their thickness is proportional to HIV-1 subtype C migration rate between state capitals as measured by BayesTraits. HIV-1 subtype C migration rate from Porto Alegre to Curitiba was more than 100 fold smaller than any of the others and is not represented in this picture.

Estimation of viral movement among localities with BayesTraits points to the role of Florianópolis as an important hub of subtype C dissemination in southern Brazil, both receiving and sending viral lineages to the other cities. The highest viral migration rate detected in our study was from Curitiba to Florianópolis (2370.1) followed by Florianópolis to Porto Alegre (956.2), Porto Alegre to Florianópolis (143.1), Florianópolis to Curitiba (86.4), Curitiba to Porto Alegre (40.1), and Porto Alegre to Curitiba (0.2) (Table 2 and Figure 4). As a possible driving force for this circulation, routine travel among analyzed states [41] was fitted to the migration rate estimates and found to be positively correlated (*r* ^2^ = 0.64), albeit not completely (Table 2 and Figure 4). Interestingly, when the viral movement rates between Santa Catarina and Rio Grande do Sul (SC to RS [956.18] and RS to SC [143.07]) were removed the correlation coefficient increased considerably to around 0.93, albeit with reduced points to determine a line. Notably, Rio Grande do Sul receives much more HIV lineages and sends much less than would be expected according to routine travel among the states.

**Table 2:** Viral migration rates and routine traffic of people within the South Brazilian region.

Origin	Destination	Viral migration rates^a^	Routine traffic (people×trip/1,000)^b^
RS - Porto Alegre	SC - Florianópolis	143.07	409
	PR - Curitiba	0.17	59
SC - Florianópolis	RS - Porto Alegre	956.18	34
	PR - Curitiba	86.37	267
PR - Curitiba	RS - Porto Alegre	40.11	42
	SC - Florianópolis	2370.10	618

a Viral migration rates amongst state capitals according to BayesTraits.

b Routine traffic amongst states according to estimations of the Public Ministry of Tourism for the year 2007 [41]. RS: Rio Grande do Sul. SC: Santa Catarina. PR: Paraná.

## Discussion

This study supports the notion that the HIV-1 subtype C epidemic affecting the south Brazilian region resulted from a single founder event, consistent with previous findings [19], [20], [21]. While a previous Bayesian phylogeography study assigned nearly equal posterior probability to either Paraná or Rio Grande do Sul as the location of such a founder event [22], our phylogeographic reconstruction place the root of the Brazilian subtype C clade at the city of Curitiba (Paraná) with high support (*PP*≥0.96). Differences in the Brazilian and/or African subtype C sequences used in each study may have a great influence in the exact position of the root of the Brazilian subtype C clade. Because we only included sequences retrieved from the southern state capitals, it will be important to test whether the support of Curitiba (Paraná) as the entrance point of subtype C is maintained or not after addition of new sequences sampled at other Brazilian cities.

After its introduction into Brazil, the subtype C clade was rapidly disseminated through the south region [42]. The work of Veras *et al* [22] points that Paraná has been the main hub of such dissemination, continuously exporting viruses to both southern neighboring states; while a minor viral gene flow from Rio Grande do Sul to Santa Catarina was also evident. Our reconstruction of the viral dispersal pattern reinforces Paraná as an important source of subtype C spread throughout the south region and rejects the hypothesis that the subtype C epidemic has been mainly spreading from the southern-most state of Rio Grande do Sul to the other Brazilian states. However, our analysis suggests that Santa Catarina has also played a key role in the southward virus spread. The inferred viral flux between Curitiba and Porto Alegre was much lower than that detected between Curitiba and Florianópolis, or between Florianópolis and Porto Alegre. Thus, Florianópolis (Santa Catarina) occupies an intermediate geographic position and seems to act as a staging post in the subtype C dissemination between Curitiba (Paraná) and Porto Alegre (Rio Grande do Sul).

Florianópolis is the most important resort locality in the region and a partial correlation of routine travel patterns and HIV-1 movement was found in our study. The most intense routine travel and the highest viral migration rates were from Paraná (Curitiba) to Santa Catarina (Florianópolis). At the other extreme, Paraná and Rio Grande do Sul displayed the less frequent routine travel and also the lowest viral migration rates within the region. People mobility, however, may only explain part of the subtype C dispersal dynamics between southern Brazilian states. This seems to be particularly evident for Rio Grande do Sul that receives much more subtype C lineages and sends much less than would be expected according to routine travel among the states. No relationship between the estimated subtype C migration rates and the size of the AIDS epidemic nor the subtype C prevalence was observed. Porto Alegre has the larger number of HIV-1 positive patients (Figure 4), but exhibits a negative net viral flux to both Curitiba and Florianópolis. On the other hand, although the prevalence of subtype C in Santa Catarina (50–80%) is much higher than in Paraná (20–30%), the estimated viral migration rate from Florianópolis to Paraná was about three times lower than the opposite viral flux.

While the previous work of Veras *et al* [22] described an homogenous subtype C epidemic across the south region of Brazil, our analyses of metapopulation structure revealed a significant subdivision of the subtype C strains among the Brazilian cities analyzed. A large proportion (67%) of subtype C infections in Porto Alegre appeared to be the result of the *in situ* dissemination of a single local lineage, here called BR-PA, which was probably introduced from Santa Catarina. Of note, previous studies have shown a high prevalence (10–35%) of the Circulating Recombinant Form 31_BC in Porto Alegre [13], [14], [15], an HIV variant rarely found (<4%) in Santa Catarina [14], [17], [18] and apparently absent in Paraná [7], [9], [10]. These results support the notion that expansion of local HIV-1 clades could be a quite common phenomenon in Porto Alegre. Despite the intense viral movement between Florianópolis and Curitiba, the null hypothesis of panmixis was also rejected for these two Brazilian cities; thus confirming the non-random distribution of HIV-1 clades across the southern region.

In conclusion, the results presented here suggest that the subtype C epidemic spreading in the southern region of Brazil was initiated by the introduction of a single founder strain probably through Paraná. This subtype C variant was rapidly disseminated to the other southern states and during this process, Florianópolis (Santa Catarina) acted as an important staging post between Curitiba (Paraná) and Porto Alegre (Rio Grande do Sul), both receiving and sending viral lineages to the neighboring cities. Meanwhile, the direct viral migration flow between Curitiba and Porto Alegre seems to be much lower. The current subtype C diversity in the south Brazilian region has also been shaped by the local dispersion of a limited set of founding strains, particularly in the southern-most capital. These data provide a more comprehensive understanding of the spatial dynamics of the subtype C epidemic spreading in southern Brazil and may be helpful to guide HIV prevention strategies in this region of the country.
